# The rational design of a Au(I) precursor for focused electron beam induced deposition

**DOI:** 10.3762/bjnano.8.274

**Published:** 2017-12-20

**Authors:** Ali Marashdeh, Thiadrik Tiesma, Niels J C van Velzen, Sjoerd Harder, Remco W A Havenith, Jeff T M De Hosson, Willem F van Dorp

**Affiliations:** 1 Zernike Institute for Advanced Materials, University of Groningen, Nijenborgh 4, 9747 AG Groningen, Netherlands; 2 Department of Chemistry, Faculty of Science, Al-Balqa’ Applied University, Salt, Jordan; 3 Stratingh Institute for Chemistry, University of Groningen, 9747 AG Groningen, Netherlands; 4 Inorganic and Organometallic Chemistry, Friedrich-Alexander Universität Erlangen-Nürnberg, Egerlandstr. 1, 91058 Erlangen, Germany; 5 Department of Inorganic and Physical Chemistry, University of Ghent, B-9000 Ghent, Belgium; 6 Uniresearch B.V., 2628 XG Delft, Netherlands

**Keywords:** crystallography, focused electron beam induced processing, gold chemistry, precursor design

## Abstract

Au(I) complexes are studied as precursors for focused electron beam induced processing (FEBIP). FEBIP is an advanced direct-write technique for nanometer-scale chemical synthesis. The stability and volatility of the complexes are characterized to design an improved precursor for pure Au deposition. Aurophilic interactions are found to play a key role. The short lifetime of ClAuCO in vacuum is explained by strong, destabilizing Au–Au interactions in the solid phase. While aurophilic interactions do not affect the stability of ClAuPMe_3_, they leave the complex non-volatile. Comparison of crystal structures of ClAuPMe_3_ and MeAuPMe_3_ shows that Au–Au interactions are much weaker or partially even absent for the latter structure. This explains its high volatility. However, MeAuPMe_3_ dissociates unfavorably during FEBIP, making it an unsuitable precursor. The study shows that Me groups reduce aurophilic interactions, compared to Cl groups, which we attribute to electronic rather than steric effects. Therefore we propose MeAuCO as a potential FEBIP precursor. It is expected to have weak Au–Au interactions, making it volatile. It is stable enough to act as a volatile source for Au deposition, being stabilized by 6.5 kcal/mol. Finally, MeAuCO is likely to dissociate in a single step to pure Au.

## Introduction

Electron microscopes, typically used for imaging and analysis, can be turned into a platform for nanoscale chemical synthesis using electron beam induced chemistry. The electron beam can act as a pen or an eraser on any solid sample, using a technique called focused electron beam induced processing (FEBIP) [1–3]. In the case of writing a precursor provides the ink, in the case of etching a precursor enables the removal of sample material. The precursors are usually gaseous, although they can also be liquid [4–5]. In the case of gaseous precursors, the gas is delivered to the sample through a gas injection system. The precursor molecules adsorb on the sample surface, and locally, where the beam interacts with the sample, electrons induce the scission of bonds in the precursor molecules [6].

**Figure 1:**
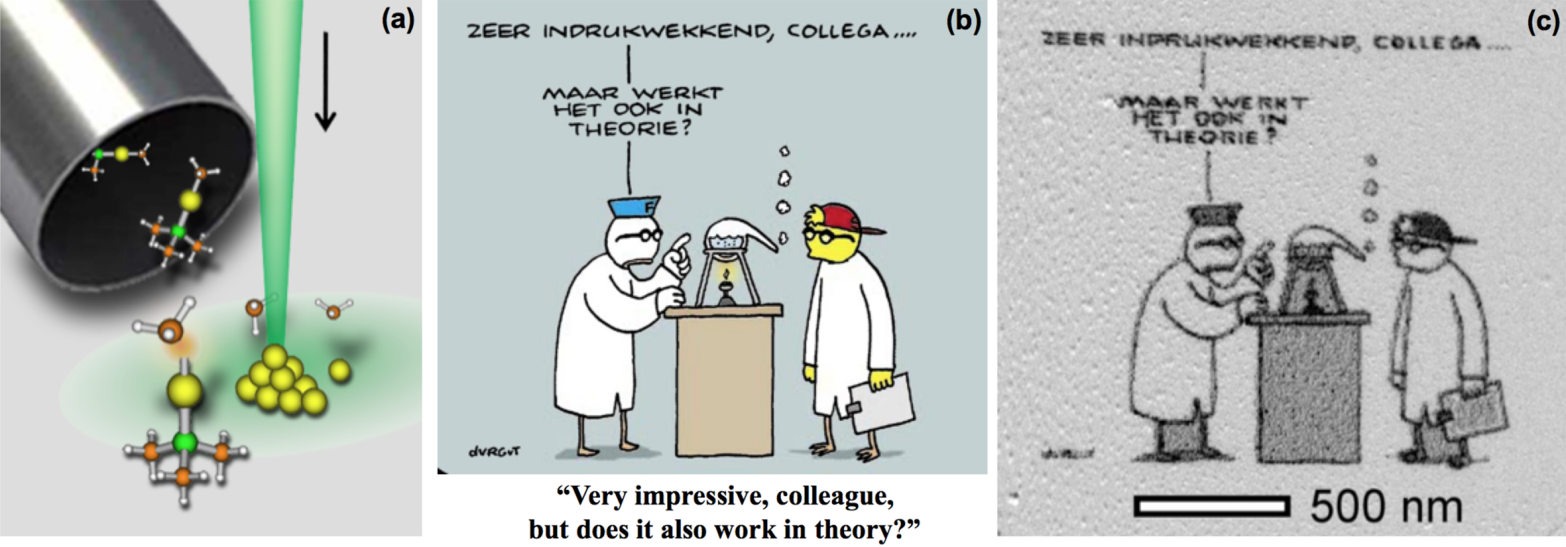
(a) A schematic drawing of the focused electron beam induced deposition. (b) A Fokke and Sukke cartoon. Reproduced with permission of Reid, Geleijnse & Van Tol. (c) The cartoon in panel (b) written on an electron-transparent membrane, using a scanning transmission electron microscope and W(CO)_6_ as precursor. The pattern consists of tungsten-containing dots of about 3 nm in size.

Figure 1a shows the deposition process. The cartoon in Figure 1b and the corresponding electron micrograph in Figure 1c show that any pattern can be written on a sample using FEBIP. The pattern is written in a scanning transmission electron microscope using W(CO)_6_ as precursor. The pixels in Figure 1c are tungsten-containing dots of about 3 nm in size.

FEBIP is an advanced and well-established technique for modifying samples on the nanometer scale. Depending on the precursor type it is possible to deposit metals (e.g., Co [7–8], Fe [9–10], Au [11–12], Pt [13–14]), insulators (e.g., SiO*_x_* [15]) and alloys (e.g., AuAg and AuPt [4]). Materials such as Si, SiO_2_, Si_3_N_4_, Cr, Ti and TaN can be etched [16]. As it is damage-free, it is used for repairing the masks for ultraviolet and extreme ultraviolet lithography [17–18], which is a major industry. FEBIP also enables the prototyping of 3D structures, such as AFM tips [19] and photonically active components [20], and the direct contacting of nanowires [21]. Furthermore, as electron beams can be focused to sub-nanometer-sized spots, the reactions can be limited to very small areas. Features as small as 0.6 nm have been written using FEBIP [22] and the deposition can be followed molecule by molecule [23].

One of the main challenges is to develop dedicated FEBIP precursors [24]. The high-energy electrons in the focused beam (typically 1–15 keV) induce reactions through ionization processes, such as dissociative electron attachment and dissociative ionization [6,25–26]. While ionization reactions can be very selective [27], they are often inefficient in removing the ligands that are currently used to make the precursor molecules volatile [28–30]. The decomposition of the precursor is then incomplete, leaving large parts of the ligand structure on the surface. This leads to for instance low metal content and poor electrical conductivity [31]. Many applications require pure metal deposition, and widespread use by non-expert users asks for a simple and fast process. While purification is possible by adding gases [32–36] or post-treatment [37], these methods only work for selected precursors and selected applications.

Designing FEBIP precursors is not straightforward, as they have to meet many requirements. They need to have tailor-made dissociation behavior, high volatility, long shelf life and they should be preferably non-hazardous and non-corrosive. The challenge is to develop a precursor that is sufficiently reactive to yield the desired reaction product in a single step, but not so reactive that it dissociates unselectively or has a short shelf life. It has already been determined that electrons cannot remove large ligands. Examples are the cyclopentadienyl ligand in trimethyl(methylcyclopentadienyl)platinum(IV) ((MeCp)PtMe_3_) [29,31], or the acetylacetonate ligand in dimethyl(acetylacetonate)gold(III) ((acac)AuMe_2_) [30], leaving the majority of the carbon in the deposit. In contrast, small groups such as halides, CO and (a single) methyl ligand can be removed [29]. Successful examples of FEBIP precursors are Co_2_(CO)_8_ [38–40], Fe(CO)_5_ [41–43] and HFeCo_3_(CO)_12_ [44]. These precursors yield deposits with a high metal content, given the right deposition conditions. W(CO)_6_ is basically too stable, resulting in a high contents of C and O in the deposit [28]. Ni(CO)_4_ on the other hand is too instable while being extremely flammable and highly poisonous. Similar to the extensive development of resists and processes for electron beam lithography, a significant research effort is required to design precursors dedicated to FEBIP.

In this paper we focus on the design criteria for Au precursors. High-purity Au deposits are of interest for many applications, such as the directed self-assembly of functional organic molecules [45], seeds for the growth of nanorods or nanotubes [46] and for plasmonics [47]. Two Au(I) compounds have been used for the deposition of pure gold. Utke et al. successfully used ClAuPF_3_ as a precursor [48] and, more recently, Mulders et al. obtained similar results using ClAuCO [11]. Similarly, experiments with PtCl_2_(CO)_2_ showed a route to deposit pure Pt [49]. While ClAuPF_3_ and ClAuCO yield deposits of high purity, they are highly unstable and decompose at room temperature with a half-life time of the order of 1 h under experimental conditions. For example, Mulders et al. report that ClAuCO releases large amounts of CO [11], while ClAuPF_3_ is also unstable [50]. In a previous study, we have explored additional Au(I) complexes. ClAuPMe_3_ appears to be relatively stable, but non-volatile [12]. MeAuPMe_3_ has been used for chemical vapor deposition (CVD) [51–52] and can be used for FEBIP. However, the electron-induced dissociation is incomplete, with just a single methyl ligand being removed [12].

The studies of Au(I) compounds that have been made so far have raised several questions. How do the ligands determine stability, shelf life and volatility? What is the origin of the short lifetime of ClAuCO in vacuum? Why is MeAuPMe_3_ volatile, while ClAuPMe_3_ is not? And can we, based on the results we have, come to a rational design of a Au precursor with the desired properties?

**Figure 2:**
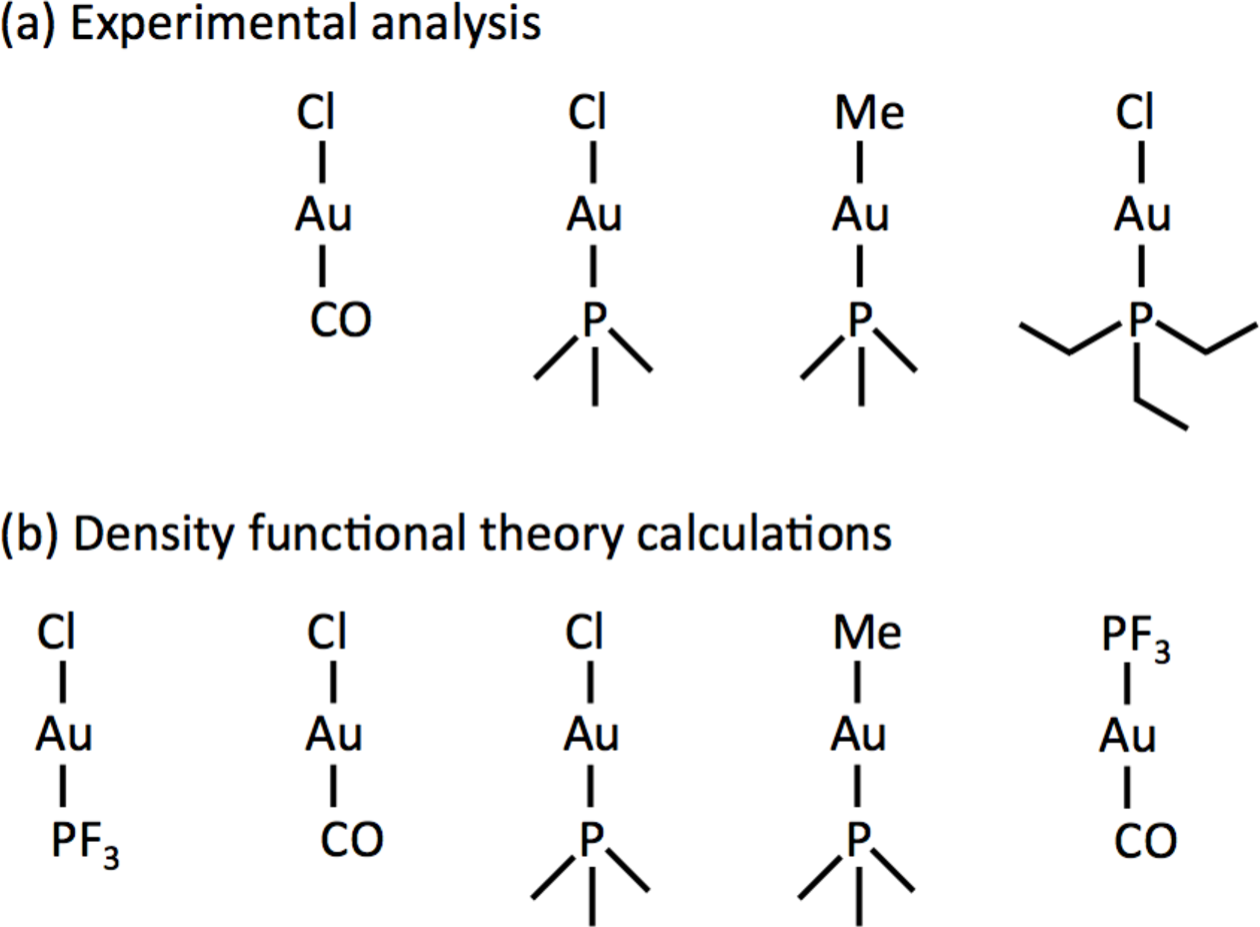
The Au(I) precursors that were studied (a) experimentally and (b) using density functional theory calculations.

In this paper we present a thorough study of organometallic gold precursors for FEBIP. Three Au(I) complexes, ClAuCO, ClAuPMe_3_ and MeAuPMe_3_, are characterized using scanning electron microscopy. The crystal structure of MeAuPMe_3_ was determined using single crystal X-ray diffraction and compared with a range of Au complexes. We combine these results with density functional theory calculations of ClAuCO, ClAuPF_3_, CF_3_AuCO, ClAuPMe_3_ and MeAuPMe_3_. The complexes are shown in Figure 2. Combining these experimental and theoretical datasets we elucidate the design rules for gold precursors. Finally, we propose a Au(I) compound for high-purity gold deposition.

## Results and Discussion

### Crystal structures of Au complexes

The stability of FEBID precursor molecules in Figure 2 strongly depends on the stability of the metal–ligand bond. Apart from that, intermolecular Au–Au interactions (aurophilicity) may have a large influence on chemical stability and volatility. The crystal structure of the new precursor in FEBID, MeAuPMe_3_, is discussed in context with a larger group of gold complexes that either contain Au–CO or Au–PX_3_ bonds. In addition, the aurophilicity in a set of Au complexes is compared.

**Figure 3:**
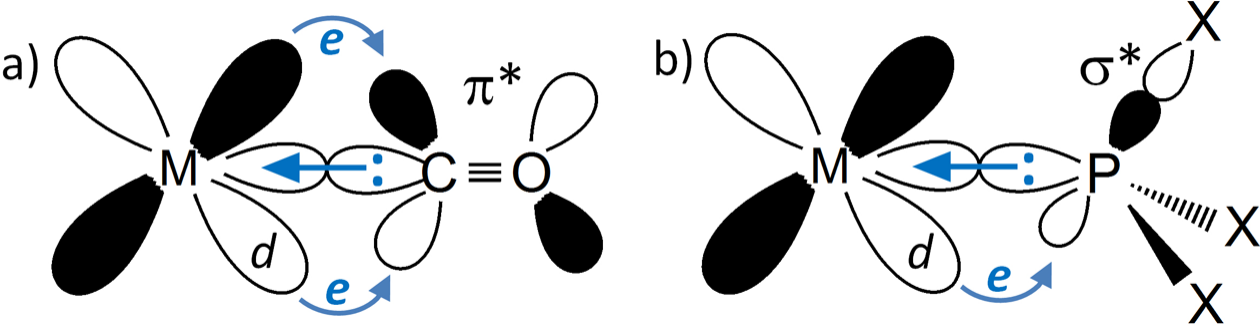
Synergistic backbonding model [53] for (a) M–CO and (b) M–PX_3_ complexes.

The bond between Au (or any other transition metal) and CO is historically explained by the synergistic backbonding model (Figure 3a) [53]. The free electron pair of the C atom can be donated into an empty orbital of the metal. Vice versa, electrons from partially filled d-orbitals can be donated back into the empty π*-orbital of CO, which has the right symmetry for overlap. The C→metal donor bond lowers the electron density from a molecular orbital that is slightly C–O anti-bonding and leads to a strengthening of the C–O bond and a shift of the C–O stretching frequency in the infrared spectrum from 2143 cm^−1^ (free CO) to higher values [54]. On the other hand, backbonding increases the electron density in the π*(C–O) orbital and causes a weakening of the C–O bond, which results in lower C–O stretching frequencies in the infrared spectrum. Since CO complexes generally show absorptions at lower frequencies (below 2143 cm^−1^) it is suggested that the backbonding is more important than the C→metal donor bond.

Early transition metals have a low electronegativity and therefore generally appear as d^0^-complexes with the metal atom in its highest oxidation state (e.g., Y(III), Ti(IV)). Consequently, there are no electrons available for backbonding and M–CO bonds are weak. There are hardly examples of CO complexes of group 3 and 4 metals of high oxidation state. Early transition metals that appear in a highly unstable low oxidation state, such as Ti(II), may form strong bonds to CO because the d-electrons that are left, are very weakly bound and can be used in strong backbonding (e.g., in the d^2^-complex Cp_2_Ti(CO)_2_) [55].

Also the late transition metals bind very weakly to CO [56]. In this case there are many electrons available for backbonding, but the late transition metals are comparably electronegative, which means that d-electrons are strongly bound and backdonation is poor. For this reason, Ni(CO)_4_ is less stable than Fe(CO)_5_. The calculated energies (BP86/ECP2) for dissociation of the first CO ligand in Fe(CO)_5_ and Ni(CO)_4_ are 44.5 and 27.5 kcal/mol, respectively [57]. Stabilities also decrease down the periodic table: The calculated CO dissociation energies for complexes of the heavier noble metals Pd and Pt are low. Consequently, Pd(CO)_4_ and Pt(CO)_4_ are unstable and only exist in an inert gas matrix at lower temperatures [58].

Similarly, CO complexes of Au(I), a noble metal of high electronegativity and with the configuration d^10^, are not very stable. ClAuCO can be prepared at 110–120 °C by passing CO over AuCl_3_ in a rapid flow [59–60]. However, both Mulders et al. and Karash et al. observe that ClAuCO easily decomposes to AuCl in vacuum, releasing CO [11,60]. Preferably ClAuCO should be prepared and kept under a CO atmosphere [61]. The CO ligand acts as a σ-donor but on the account of the high electronegativity of Au, there is only little backbonding. This leads to a strengthening of the C-–O bond and an increase of the C–O stretching frequency from 2143 cm^−1^ (for free CO) to 2162 cm^−1^ (for ClAuCO in CH_2_Cl_2_) [62]. For this reason ClAuCO can, similarly to Cu or Ag carbonyl complexes, be categorized as an unusual “non-classical metal-carbonyl complex” [63].

Also, a large variety of complexes with Au–PX_3_ bonds are known (X relates to any organic or inorganic moiety). Phosphines are excellent ligands for transition metals and, similar as in M–CO complexes, their bonding can be described with a synergistic backbonding model. In this case, however, the main bond is formed by the donation of the phosphor lone pair of electrons into an empty orbital of the metal. The metal→P backbonding of d-electrons into the empty σ*_P–X_ orbital on the phosphine is weak and of minor importance (Figure 3b). Whereas PMe_3_ is a strong electron-pair donor (the Me groups are electron-releasing), the PF_3_ ligand with electron-withdrawing F substituents is a very weak donor. On the other hand, the subordinate metal→P backbonding is stronger for PF_3_, which is an excellent acceptor on due to its low-lying σ* orbitals.

The electron-donor abilities of phosphine ligands are quantified by the Tolman electronic parameter [64]. The stretching frequency of CO in Ni(CO)_3_PX_3_ complexes is taken as a measure for the electron density on the metal and is directly related to the electron-donor abilities of the PX_3_ ligand (low C–O stretching frequencies relate to strong donor abilities of PX_3_). The large difference between ν_CO_ for PMe_3_ (2064 cm^−1^) and PF_3_ (2110 cm^−1^) reflects their strongly different donor abilities (PMe_3_ >> PF_3_).

**Figure 4:**
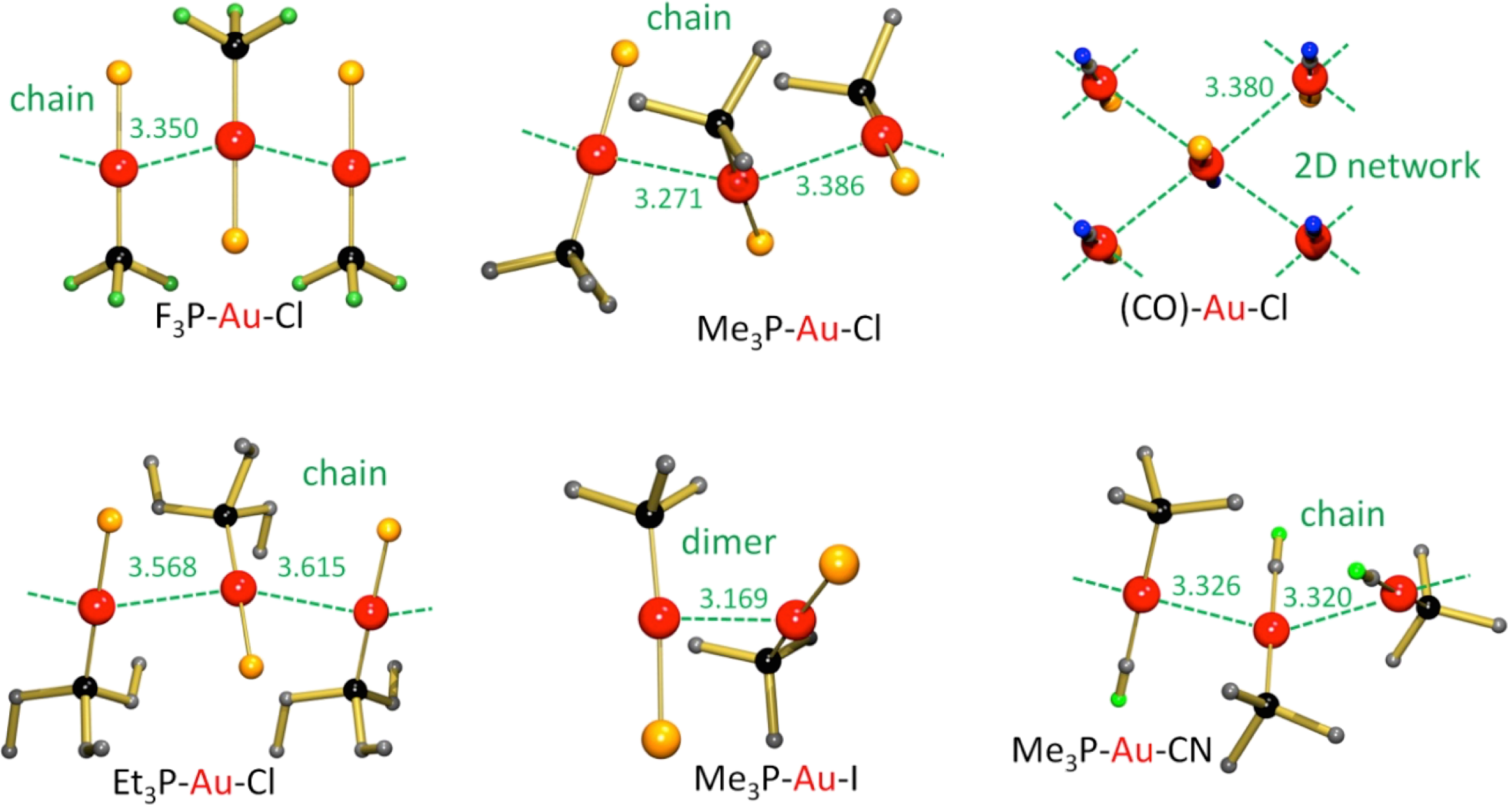
Crystal structures with aurophilic interactions. The green dashed lines indicate the Au–Au interactions, distances are given in angstroms. (a) ClAuPF_3_ [69,79], (b) ClAuPMe_3_ [68], (c) ClAu(CO) [71], (d) ClAuPEt_3_ [78], (e) IAuPMe_3_ [58,80], (f) CNAuPMe [81].

The observed differences in stability or volatility may be due to aurophilicity, a well-known phenomenon in Au(I) chemistry [65–66]. Aurophilicity is the unusual tendency of gold compounds with closed-shell Au(I) atoms ([Xe]4f^14^5d^10^6s^0^) to form weak Au–Au bonds (Figure 4). Two Au atoms can be considered to interact when the Au–Au distance is shorter than 3.7 Å, i.e., twice the van der Waals radius for Au. The aurophilic bond generally displays lengths of circa 3.0–3.4 Å and bond strengths of circa 7–12 kcal/mol [65]. Relativistic effects amount to 28% of the binding energy and originate almost exclusively from the relativistic expansion of the gold d-shell [67].

Au–Au interactions seem to be governed by sterics: The presence of large ligands impedes any possible interaction. However, there seems to be no relationship with the electron-donating capability of the phosphine ligand and the Au–Au distance. The complexes ClAuPMe_3_ and ClAuPF_3_ (shown in Figure 4) show aurophilic interactions over a similar distance [68–69]. It was claimed that hardness/softness of ligands could have an effect on aurophilicity [70], but this was later rejected by Schmidbaur and co-workers [68]. The complex ClAuCO shows four short Au–Au bonds per Au atom and should, therefore, be regarded as being strongly bound by aurophilic interactions [71]. This can be attributed to the small size and needle-like coordination of the CO ligand. Figure 4 shows that ClAuCO crystallizes as a 2D polymeric structure. It seems that the strength of aurophilic interactions is mainly determined by the size and form of the ligands. For instance, of the complexes shown in Figure 4, ClAuPEt_3_ has relatively large ligands and relatively long Au–Au bonds. The electronic or soft/hard properties of the ligands appear not to affect aurophilicity very much, as the sequence of ClAuPMe_3_, IAuPMe_3_ and CNAuPMe_3_ shows. Quantification of aurophilicity is difficult. One should not simply correlate it to the Au–Au distances, but also consider the dimension of the network and the number of Au–Au interactions. A short single Au–Au interaction is not necessarily stronger than a 2D network with four longer aurophilic interactions. We have evaluated the Au–Au distances and the type of network to classify the strength of aurophilic interactions in the three categories: weak, medium and strong (Table 1).

**Table 1:** Aurophilic Au–Au interactions in complexes of the type (ligand)–Au–Cl and Me_3_P–Au–X.

complex	ref.	Au–Au distance (Å)	type	strength	melting point (°C)

(Ligand)-Au-Cl complexes					

Et_3_P–Au–Cl	[72–73]	3.592(5)	chain	weak	160–170
Me_3_P–Au–Cl	[68]	3.338(1)	chain	medium	215–228
F_3_P-Au-Cl	[69,74]	3.350(1)	chain	medium	45^a^
(CO)–Au–Cl	[71]	3.380(3)	2D polymer	strong	247–253^a^

Me_3_P–Au–X complexes					

Me_3_P–Au–Cl	[68]	3.338(1)	chain	medium	215–228
Me_3_P–Au–I	[58,75]	3.169(1)	dimer	medium	209–214
Me_3_P–Au–CN	[70,76]	3.289(2)	chain	medium	197–199^a^
Me_3_P–Au–Me	this work, [52]	3.3602(5)	dimers/monomers	weak	70–71

^a^Decomposition temperature.

**Figure 5:**
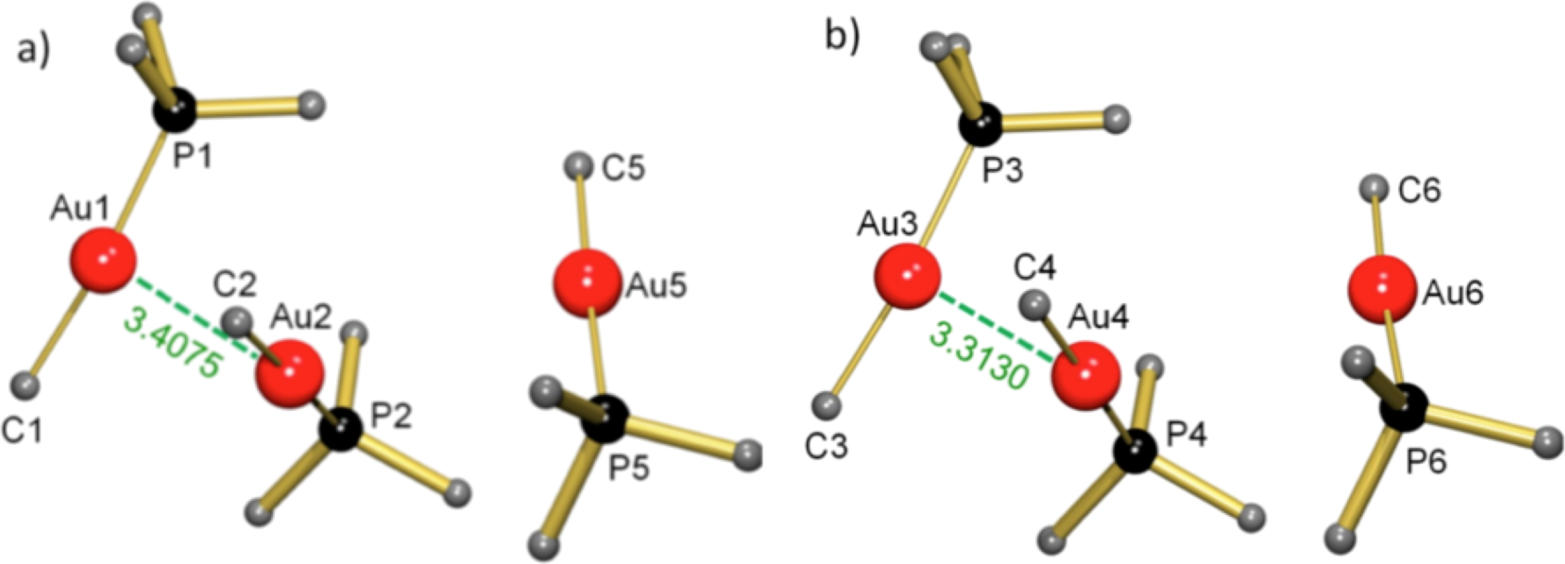
Crystal structure of MeAuPMe_3_. Two groups of three molecules (a, b) have a very similar but not equal packing and differ by a slightly different orientation.

We have determined the crystal structure of MeAuPMe_3_. This complex crystallizes in the triclinic space group *P*−1 with six molecules in the asymmetric unit (Figure 5). The six independent molecules can be separated in two groups of three that have very similar but not equal packing and that differ by a slightly different orientation and distance in respect of each other (no higher symmetry could be found). The average Me-Au bond of 2.067(5) Å (range: 2.063(5)–2.075(5) Å) is comparable to the C-Au bond lengths of 2.07(1) Å in Ph-C≡C-Au-PMe_3_ and 2.03(3) Å in (CN)AuPMe_3_. The Au-P bond of 2.287(1) Å (range: 2.283(1)–2.292(2) Å) is at the higher end of the range observed in Au-PMe_3_ complexes summarized in Table 1 (2.233(3)–2.276(6) Å).

**Figure 6:**
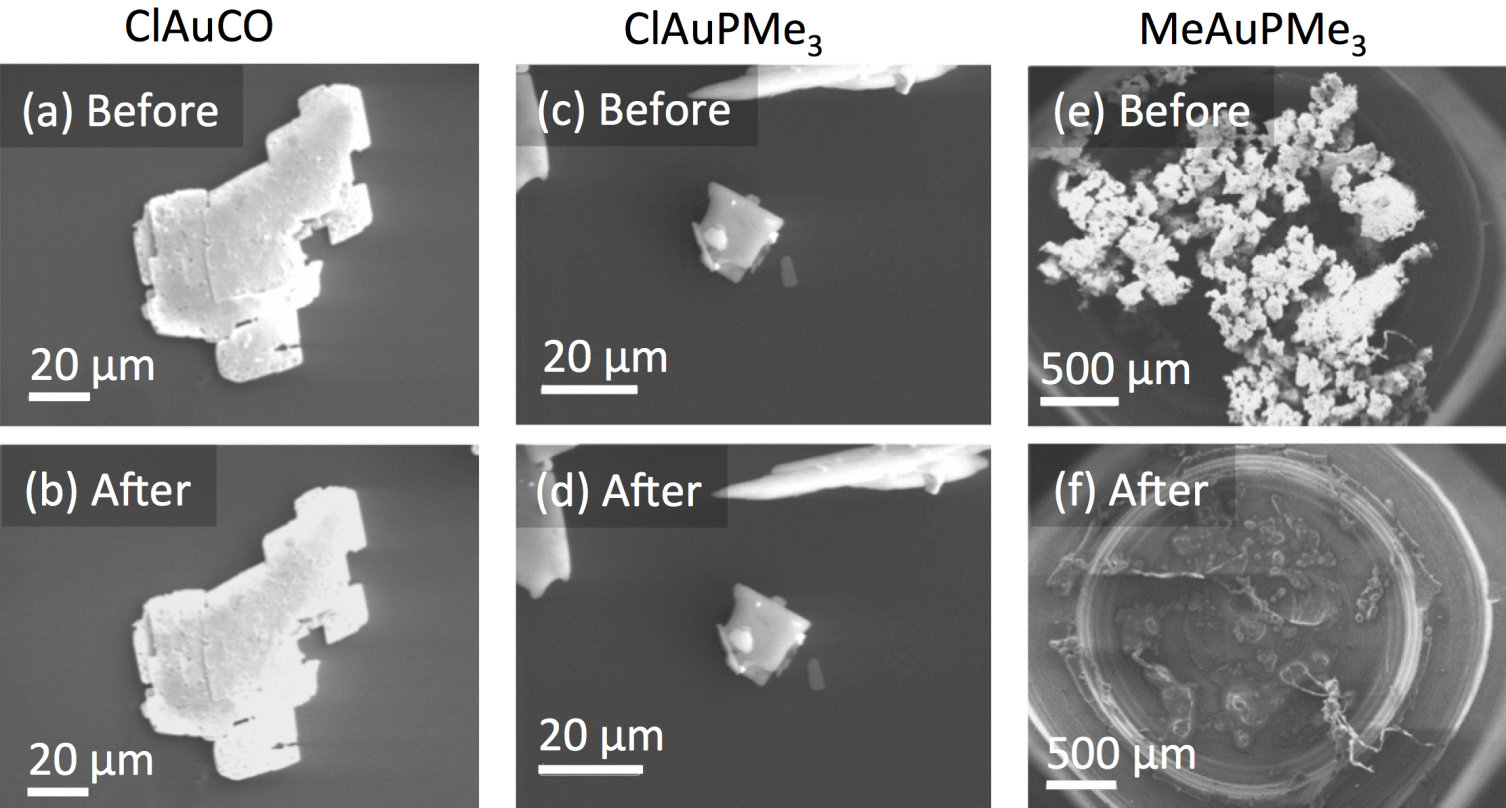
ClAuCO (a) before and (b) after 12 h in vacuum. No changes were observed. For ClAuPMe_3_ also no changes were observed (c) before and (d) after 12 h in vacuum. MeAuPMe_3_ (e) was found to sublime within about 20 min (f).

Of major interest are the aurophilic interactions. Four out of the six molecules in the asymmetric unit form weakly bound dimers with rather long Au∙∙∙Au interactions of 3.3130(4) Å and 3.4073(5) Å (average: 3.33602(5) Å). In contrast, two molecules in the unit cell show Au∙∙∙Au contacts > 4.0 Å and should basically be considered monomeric. The clearly less pronounced formation of aurophilic bonds in MeAuPMe_3_ compared to ClAuPMe_3_ should likely be explained by a difference in electronic factors instead of sterics: the Me group has the same size as a Cl group but an opposite electronic influence. Weak aurophilic interactions in MeAuPMe_3_ are in good agreement with the high volatility of MeAuPMe_3_ compared to ClAuPMe_3_ (Figure 6).

### Electron microscopy

Similar to the analysis in [12] crystals of the compounds (2 to 50 μm in size) were observed in the electron microscope. Samples were introduced into the SEM supported on an Si wafer and free of water and oxygen. The samples were imaged directly after loading and after 12 h in vacuum. As Figure 6 shows, no significant changes were observed for ClAuCO and ClAuPMe_3_. The same behavior was observed for ClAuPEt_3_ crystals (not shown). MeAuPMe_3_ was found to sublime within about 20 min in vacuum.

**Figure 7:**
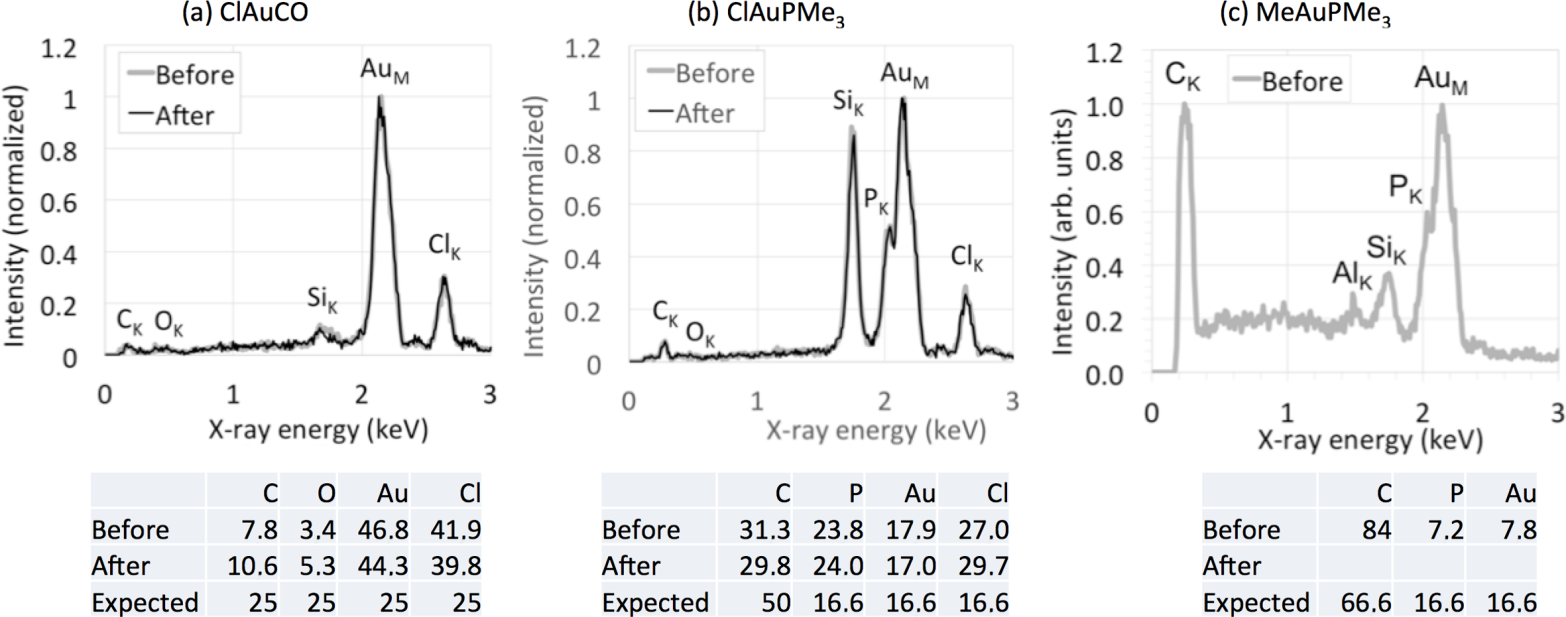
Compositional analysis of Au(I) complexes using EDS. (a) For ClAuCO the Au/Cl ratio was about 1:1, and little C and O was detected. (b) For ClAuPMe_3_ approximately the correct stoichiometry was detected, both before and after 12 h in vacuum. (c) For MeAuPMe_3_ more C was detected than expected, which we attribute to remnants of silicone grease used during the synthesis.

The composition of the crystals was analyzed using energy-dispersive X-ray spectroscopy (EDS). It has to be noted that, with an anisotropic material distribution of relatively small crystals sitting on a supporting material, the reliability of quantitative EDS is limited. It is also known that crystals of such organometallic complexes can be sensitive to electrons [12]. However, in these experiments we do consider EDS to be very useful. Firstly, the EDS measurements are merely intended to see whether the compounds auto-decompose in vacuum, as the material purities have already been confirmed with other techniques. Secondly, EDS measurements in the SEM do enable us to analyze all relevant elements in the crystals. And finally, potential (concurrent) changes in morphology and composition can be observed directly under the relevant conditions, i.e., in high vacuum. While it means that extra care has to be taken (and margins on the quantification have to be added) when interpreting the EDS results, the results do reveal the stability of the complexes. The results are shown in Figure 7. For ClAuCO only decomposed material was found, and we consistently did not detect any trace of ClAuCO. The Au/Cl ratio was about 1:1, taking into account the experimental errors, and little C and O was detected (Figure 7a). That the composition basically did not change during the 12 h in vacuum, strongly suggests that AuCl has formed before/during entering the sample into the microscope. We therefore conclude that ClAuCO decomposes very quickly to AuCl. This conclusion is consistent with earlier reports by Karash et al. that ClAuCO decomposes rapidly in vacuum [60].

For ClAuPMe_3_ (Figure 7b), less C and more P and Cl were found than expected. We attribute this deviation from the stoichiometric composition to the anisotropic material distribution and the electron sensitivity of the crystals. Most relevant to our analysis is that there was no change in composition after 24 h in the microscope. ClAuPEt_3_ was found to behave very similarly (not shown). We conclude from these results that ClAuPMe_3_ and ClAuPEt_3_ are non-volatile and that their composition is not significantly affected by the vacuum.

For MeAuPMe_3_, the ratio between P and Au of about 1:1 is as expected. More C was found than expected. Figure 7c shows that not only C, P and Au were detected, but also Si and O. As observed earlier [12], the crystals likely contain remnants of silicone grease that was used during the synthesis. The residue that remains after 20 min consists of SiO*_x_*C*_y_*, confirming that the MeAuPMe_3_ is volatile. The Al signal and the ring structure come from the supporting Al stub.

### DFT calculations on isolated molecules

Figure 6 and Figure 7 show that ClAuCO is unstable in vacuum, decomposing rapidly to AuCl, while ClAuPMe_3_ and MeAuPMe_3_ are stable. To understand why the stability of these compounds varies so much, we have calculated the changes in Gibbs free energy (Δ*G*) for particular reactions for isolated molecules at 298 K and a pressure of 1 × 10^−4^ Pa (1 × 10^−6^ mbar). Table 2 shows the reaction energies, including those for ClAuPF_3_ (which is as unstable as ClAuCO [50]) and CF_3_AuCO. Please note that the values in Table 2 are related to thermodynamical ground states and do not represent the activation energies for decomposition.

**Table 2:** Calculated reaction energies for isolated Au(I) complexes. The values of Δ*G* are calculated for a temperature of 298 K and a pressure of 1 × 10^−4^ Pa.

		reaction	Δ*G* (kcal/mol)

ClAuCO	→ AuCl + CO	(1)	+25.5
	→ Cl + AuCO	(2)	+78.8
	→ Cl + Au + CO	(3)	+67.8
ClAuPF_3_	→ AuCl + PF_3_	(4)	+16.5
	→ Cl + AuPF_3_	(5)	+72.2
	→ Cl + Au + PF_3_	(6)	+58.8
CF_3_AuCO	→ CF_3_Au + CO	(7)	+10.1
	→ CF_3_ + AuCO	(8)	+151.4
	→ CF_3_ + Au + CO	(9)	+143.6
ClAuPMe_3_	→ AuCl + PMe_3_	(10)	+36.9
	→ Cl + AuPMe_3_	(11)	+77.9
	→ Cl + Au + PMe_3_	(12)	+79.2
MeAuPMe_3_	→ MeAu + PMe_3_	(13)	+11.6
	→ Me + AuPMe_3_	(14)	+43.3
	→ Me + Au + PMe_3_	(15)	+44.6

The most favorable reaction path for ClAuCO is the dissociation into AuCl and CO. That is consistent with our experimental results and those reported by Mulders et al. and Karash et al., who have observed the formation of AuCl and the release of CO in vacuum [11,60]. The value of Δ*G* is positive, i.e., the reaction as endergonic. The calculated reaction energy partially explains the behavior observed in the experiment, behavior that appears to be contradictory. On the one hand, solid crystalline ClAuCO decomposes so rapidly that we only detect AuCl in the electron microscope. This instability is inconsistent with a Δ*G* value of +25.5 kcal/mol. On the other hand, Mulders et al. have shown that, once ClAuCO molecules reach the gas phase, they are stable enough to travel from the precursor reservoir, through the gas injection needle to be ionized and dissociated by the electron beam [11]. The latter behavior is much more consistent with a Δ*G* value of +25.5 kcal/mol. Judging from the value of Δ*G* of +16.5 kcal/mol, ClAuPF_3_ is less stable in vacuum than ClAuCO. This might be consistent with the experimental observation that ClAuPF_3_ has a short lifetime in vacuum, but there is not enough data to further quantify that statement. CF_3_AuCO appears to be the least stable of the studied compounds, which is consistent with literature data. Martínez-Salvador et al. describe CF_3_AuCO as only stable at low temperatures and quickly darkening at room temperature, preventing elemental analysis [77]. To our knowledge there is no literature data on the stability of CF_3_AuCO in vacuum (for the solid phase nor for the gas phase). ClAuPMe_3_ is the most stable compound in Table 2, the lowest value of Δ*G* being 36.9 kcal/mol. This is consistent with our experimental observations described in Figure 5 and Figure 6. Regarding MeAuPMe_3_, the lowest value of Δ*G* is +11.6 kcal/mol for reaction 13. At least the value of Δ*G* appears to be consistent with the observations that MeAuPMe_3_ is stable enough to be used as a precursor for chemical vapor or electron-induced deposition [12,51–52].

Concluding, the DFT calculations of the ground states of isolated molecules help to explain the stability of ClAuPF_3_, CF_3_AuCO and ClAuPMe_3_. MeAuPMe_3_ appears to be stabilized by a significant activation barrier for decomposition, as it is more stable in practice than the low value of Δ*G* of +11.6 kcal/mol suggests. As for ClAuCO, the calculations appear to overestimate its stability, as it decomposes faster than the Δ*G* value of +25.5 kcal/mol would lead us to expect. From this we conclude that for ClAuCO we need to consider not only isolated molecules, but also to include interactions with neighboring molecules.

### Periodical calculations for ClAuCO

Considering the aurophilic interactions in solid ClAuCO [71], we suggest that interactions with neighbors destabilize the compound. In other words, the crystal structure opens a dissociation path to form AuCl, one that has a lower value of Δ*G* than the dissociation path for isolated molecules. To test this hypothesis, we have performed periodical calculations for ClAuCO and AuCl.

**Figure 8:**
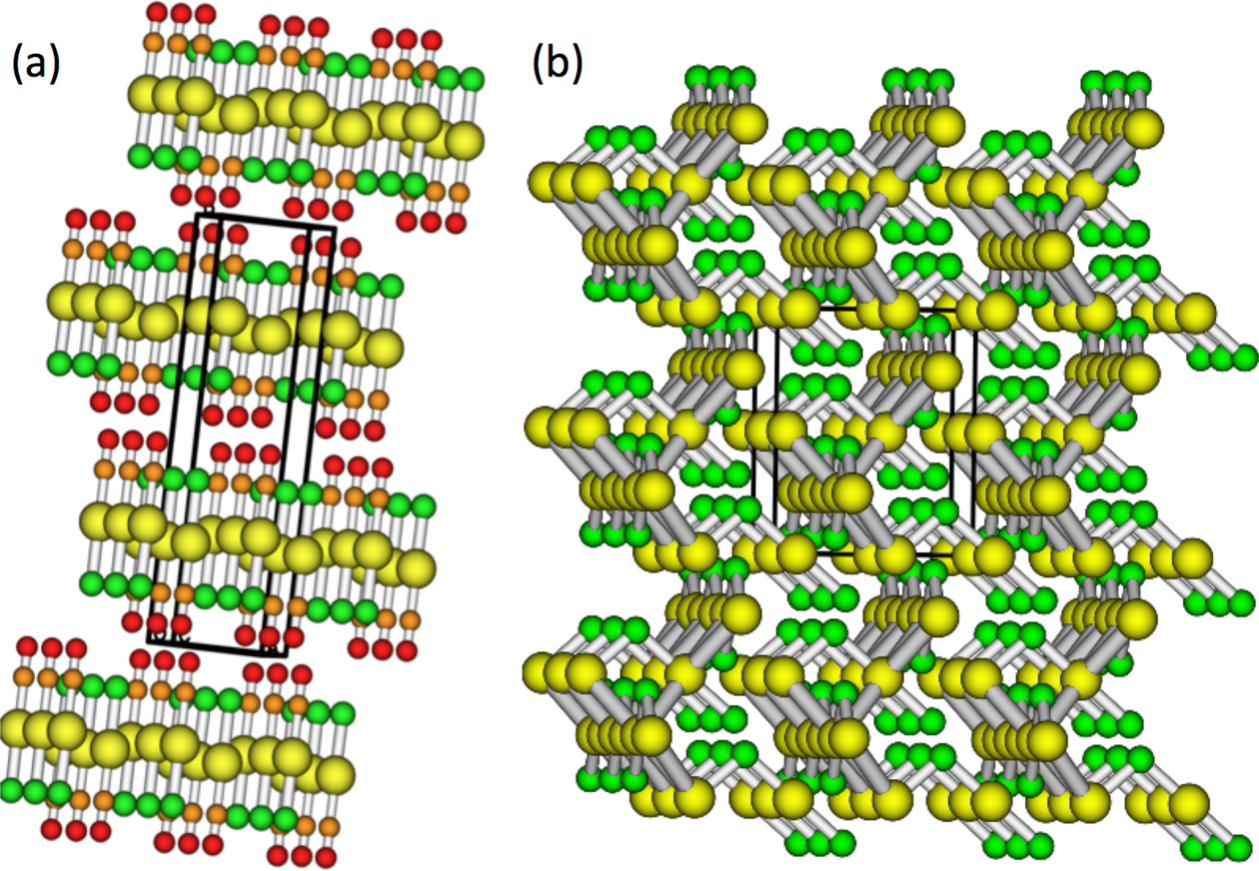
Periodic structure calculations for crystal structures of (a) ClAuCO and (b) AuCl.

Figure 8a and Figure 8b show the periodic structures for ClAuCO and AuCl, respectively. The calculated Au–Au distances are 3.41 Å, matching closely the experimental value of 3.38 Å [71]. While the value of Δ*G* for decomposing ClAuCO_(s)_ into AuCl_(s)_ and CO_(g)_ is +10.5 kcal/mol at atmospheric pressure, the value of Δ*G* drops to −1.8 kcal/mol at a pressure of 1 × 10^−4^ Pa. The negative value of Δ*G* confirms the hypothesis that aurophilic interactions with neighboring molecules destabilize the compound, and supports the experimental observations. The low value of Δ*G* of −1.8 kcal/mol explains the instability of solid ClAuCO in vacuum.

The significantly lower value of Δ*G* obtained for the solid state (+10.5 kcal/mol) compared to the gas phase value of Δ*G* of +36.75 kcal/mol at atmospheric pressure indicates a stabilization of AuCl (or destabilization of ClAuCO) due to interactions in the solid state. The further decrease in Δ*G* upon going from atmospheric pressure to vacuum is indicative for the entropic effect. Similar effects are likely to occur in ClAuPF_3_, since the value of Δ*G* for dissociation, the crystal structure and the aurophilic interactions are very similar. Experimental evidence has yet to be obtained to qualify or quantify this.

Having clarified the issues regarding the stability of the Au(I) compounds, we now focus on the volatility of ClAuPMe_3_ and MeAuPMe_3_. The trend mentioned in the theoretical considerations suggests a causal relationship between aurophilicity and volatility. Figure 6 and Figure 7 show that ClAuPMe_3_ is non-volatile, which is consistent with its strong aurophilic interactions. In contrast, MeAuPMe_3_ is volatile. So, extrapolating from the observed trend, aurophilic interactions should not be dominant in MeAuPMe_3_. This is indeed confirmed by the crystal structure of MeAuPMe_3_ (see Figure 5).

### Understanding at the molecular level

Considering the literature data, the SEM analysis, the DFT calculations and the XRD measurements, we now understand the stability and volatility of Au(I) complexes at the molecular level. Regarding ClAuCO, DFT calculations on isolated molecules show that they are stabilized by at least +25.5 kcal/mol. On the other hand, periodic DFT calculations show that strong aurophilic interactions in the crystal destabilize the compound, leading to a Δ*G* value of −1.8 kcal/mol. We therefore conclude that the lifetime of ClAuCO in vacuum is not limited by the stability of isolated molecules, but rather by the stability of solid ClAuCO. The calculations also explain why ClAuCO can be used as a FEBIP precursor. In the experiments by Mulders et al. [11], ClAuCO_(s)_ releases a small amount of ClAuCO_(g)_ molecules as it decomposes to AuCl_(s)_. Once in the gas phase, these relatively isolated molecules are stabilized by at least +25.5 kcal/mol. That enables ClAuCO_(g)_ to act as a volatile source for Au deposition.

As Mulders et al. and Wnuk et al. have shown, electrons are very efficient at removing the Cl and CO ligands [11,78], leading to high-purity Au deposits. Hence, if it were not for the destabilizing aurophilic interactions in the crystal, ClAuCO would be a very suitable FEBIP precursor.

ClAuPMe_3_ and ClAuPEt_3_ are chemically stable at room temperature and in vacuum, as demonstrated by experiments and DFT calculations. The strong aurophilic interactions in the crystal prevent the molecules from escaping to the gas phase, making the compounds non-volatile. ClAuPMe_3_ and ClAuPEt_3_ are hence not useful as FEBIP precursor.

However, replacing the Cl ligand with a Me ligand improves the volatility. MeAuPMe_3_ is chemically stable enough to act as a precursor for FEBIP (and chemical vapor deposition) and crystallizes with six molecules in an asymmetric unit. While four molecules in the unit have strong aurophilic interactions, two molecules have Au–Au distances of more than 4.0 Å. These two should basically be considered monomeric. We observe that the threshold for desorption is relatively low for these more loosely bound molecules. As some of the MeAuPMe_3_ molecules leave to the gas phase, the crystal structure is lost. Finally, all molecules can desorb. But although MeAuPMe_3_ is volatile, it is not a very good FEBIP precursor. Experimental results suggest that electrons induce the scission of the Me–Au bond [12,78], and that the PMe_3_ ligand stays on the surface. MeAuPMe_3_ therefore does not yield pure Au deposits.

### Rational design of a new precursor

Extrapolating from these insights, there are two solutions for a new Au(I) FEBIP precursor. Firstly, the destabilizing effect of aurophilic interactions in ClAuCO may potentially be reduced by preventing the formation of the ClAuCO crystal structure. In the absence of aurophilic interactions, it is likely that solid ClAuCO becomes volatile in which case it would be an ideal FEBIP precursor. Possibly the formation of the 2D polymeric structure can be prevented by forcing the condensation of ClAuCO in the nanopores of a zeolite, directly after synthesis. We expect this to be a challenging experimental route. The second solution is the rational design of a new precursor. Me ligands reduce aurophilic interactions and thereby increase the stability of Au(I) compounds. Also, low-energy electrons are efficient in breaking CO–metal and Me–metal bonds [12,78].

Based on this information, we propose MeAuCO as a gold precursor for FEBIP and expect it to have only weak Au–Au interactions in the crystal. It is therefore very likely to be volatile. We have calculated the (ground state) reaction energies for MeAuCO. As Table 3 shows, it is stabilized by at least +6.5 kcal/mol, which suggests it is stable enough to be used as a precursor. Possibly it needs to be kept at low temperatures to avoid thermal decomposition in the reservoir, but that is technically feasible. When MeAuCO is exposed to electrons, it is likely to dissociate in a single step to pure Au. The reaction fragments, CO and Me radicals, are not aggressive and do not damage either sample or microscope. Very recently, the related complex CF_3_AuCO has been isolated [77]. The target complex MeAuCO is likely less stable but may exist. Provided the compound can be synthesized [77], we expect it to be a very suitable FEBIP precursor.

**Table 3:** Calculated reaction energies for MeAuCO.

		reaction	Δ*G* (kcal/mol)

MeAuCO	→ MeAu + CO	(18)	+6.5
	→ Me + AuCO	(19)	+47.2
	→ Me + Au + CO	(20)	+39.4

## Conclusion

Electron microscopy experiments show that ClAuCO decomposes rapidly into AuCl in vacuum. This is consistent with reports in literature, where the release of CO was observed. A similarly short lifetime in vacuum was reported for ClAuPF_3_. The experimental instability of solid ClAuCO is in contradiction to DFT calculations, which show that isolated ClAuCO molecules in the gas phase are stabilized by at least +25.5 kcal/mol. Both ClAuCO and ClAuPF_3_ exhibit strong aurophilic interactions in the crystals. Periodical DFT calculations of solid ClAuCO show that these Au–Au interactions destabilize the Au–CO bond in the crystal. The value of Δ*G* for the formation of AuCl is lowered to −1.8 kcal/mol, thereby explaining the short lifetime of solid ClAuCO in vacuum.

The complexes ClAuPMe_3_ and ClAuPEt_3_ show medium and weak Au–Au interactions, respectively. Both complexes are stable in vacuum, which is consistent with the DFT calculations, and both are non-volatile.

Experimental observations show that MeAuPMe_3_ is stable and volatile. This is consistent with the DFT calculations, which show that the complex is stabilized by +11.6 kcal/mol. XRD shows that MeAuPMe_3_ crystallizes in the triclinic space group *P*−1 with six molecules in the asymmetric unit. The six independent molecules can be separated in two groups of three that have very similar but not equal packing. Four out of the six molecules in the asymmetric unit form weakly bound dimers with Au–Au interactions. In contrast, two molecules in the unit cell show Au–Au distances above 4.0 Å and should basically be considered monomeric. These monomerically bound MeAuPMe_3_ molecules have a lower desorption energy, allowing them to leave to the gas phase. Once these MeAuPMe_3_ molecules desorb, the crystal structure is broken up, enabling all molecules to leave to the gas phase.

The precursors ClAuCO and ClAuPF_3_ are known to yield high-purity Au deposits during FEBIP. Our observations show that the crystal structure plays a dominant role in the stability and volatility of Au(I) complexes. To increase the stability and volatility, aurophilic interactions have to be reduced. Our results show that the Me group, while having the same size as a Cl group, reduces Au–Au interactions because of its opposite electronic influence. Based on these results, we come to the rational design of a Au precursor: MeAuCO. DFT calculations show that isolated MeAuCO is stable at standard FEBIP conditions.

## Experimental

### Density functional theory calculations

Calculations on the molecules were performed using the B3LYP functional with the aug-cc-pVDZ-PP and aug-cc-pVDZ basis sets (for the Au atoms and for all other atoms, respectively), with GAMESS-UK [79]. All stationary points were characterized as genuine minima through Hessian calculations (no imaginary frequencies were found). Thermodynamical properties were calculated at 298 K and a pressure of 1 × 10^−4^ Pa.

Periodic DFT (B3LYP) calculations on ClAuCO and AuCl were performed with the Crystal14 program [80], with a basis set based on the (aug-)cc-pVDZ basis sets (the diffuse s,p functions were removed to avoid linear dependencies). For gold a basis set derived from the cc-pVDZ-PP was used (without diffuse s and p functions). A shrinking factor of 8 was chosen. Full geometry optimizations and frequency calculations [81–82] including the Grimme dispersion correction were performed [83]. Thermodynamics were calculated at 298.15 K and 1.0 × 10^−9^ MPa, and 298.15 K and 0.101 MPa.

### Synthesis

MeAu(PMe_3_) was synthesized as described in [12]. The products were analyzed by C and H elemental analysis and showed satisfactory values. MeAu(PMe_3_), anal. calcd for C_4_H_12_PAu: C, 16.68; H, 4.20; found: C, 17.15; H, 4.26.

Samples were also analyzed by ^1^H and ^31^P NMR spectroscopy. MeAu(PMe_3_): ^1^H NMR (400 MHz, C_6_D_6_, 25 °C) δ 1.20 (d, ^3^*J*_HP_ = 8.5 Hz, 3H, AuCH_3_), 0.60 (d, ^2^*J*_HP_ = 8.7 Hz, 6H, PCH_3_); ^31^P NMR (162 MHz, C_6_D_6_, 25 °C) δ 11.4 (m, ^2^*J*_HP_ = 8.7 Hz, PMe_3_). These values correspond to values published earlier [84–85].

The compound was stored at 243 K in a dry N_2_ atmosphere and loaded into vacuum reservoirs, either a stainless steel reservoir or the Al crucible of an FEI gas injection system (GIS) in Ar or N_2_ atmosphere.

### Electron microscopy

Crystals of ClAuCO, ClAuPMe_3_, ClAuPEt_3_ and MeAuPMe_3_ were inserted in the sample chamber of a Philips XL30 environmental scanning electron microscope (SEM), equipped with a field-emission gun and an EDAX detector for energy-dispersive X-ray spectroscopy (EDX). The samples were inserted free of oxygen and water. The composition and morphology were characterized directly after inserting, and after 12 h in high vacuum.

### X-ray diffraction

High-quality single crystals of MeAuPMe_3_ were grown by recrystallization of micro-crystalline material: slow evaporation of the solvent from a diethylether solution in the inert atmosphere of a glovebox gave single crystals between 0.1 and 0.5 mm. The crystals were measured on a SuperNova diffractometer (Rigaku-Oxford diffractions) with Mo and Cu microsources and an Atlas S2 detector. The crystals were covered with paraffin oil in a glovebox, mounted on a loop and then cooled to −100 °C. The crystal structure was solved by Direct Methods (SHELXS-97) [86] and refined against F^2^ with SHELXL-97 [86]. All geometry calculations, checks for higher symmetry and graphics were performed with PLATON [87]. The hydrogen atoms have been placed on calculated positions and were refined isotropically in a riding mode.

Crystal data: measurement at −100 °C (Mo Kα), formula (C_4_H_12_AuP)_6_, triclinic, *a* = 11.4591(5) Å, *b* = 13.5930(5) Å, *c* = 16.0117(7) Å, α = 107.433(1)°, β = 90.097(1), γ = 114.317(1), *V* = 2145.83(16) Å^3^, space group *P*−1, *Z* = 2, ρ_calc_ = 2.675 g·cm^−3^, μ(Mo Kα) = 20.671 mm^−1^, 50600 measured reflections, 12067 independent reflections (*R*_int_ = 0.038), 10026 reflections observed with *I* > 2*σ*(*I*), θ_max_ = 31.0°, *R* = 0.0272, *wR2* = 0.0535, GOF = 1.06, 349 parameter, min/max residual electron density −1.84*e*·Å^−3^/+2.02*e*·Å^−3^.

Crystallographic data (excluding structure factors) have been deposited with the Cambridge Crystallographic Data Centre as supplementary publication no. CCDC 1492142. Copies of the data can be obtained free of charge on application to CCDC, 12 Union Road, Cambridge CB21EZ, UK (fax: (+44)1223-336-033; email: deposit@ccdc.cam.ac.uk)
